# Mercury removal during growth of mercury tolerant and self-aggregating *Yarrowia* spp.

**DOI:** 10.1186/s13568-016-0271-3

**Published:** 2016-10-13

**Authors:** Ganiyu Oladunjoye Oyetibo, Keisuke Miyauchi, Hitoshi Suzuki, Ginro Endo

**Affiliations:** 1 Department of Civil and Environmental Engineering, Faculty of Engineering, Tohoku-Gakuin University, 1-13-1 Chuo, Tagajo, Miyagi 985-8537 Japan; 2 Department of Microbiology, Faculty of Science, University of Lagos, Akoka, Yaba, Lagos, Nigeria; 3 Department of Electronic Engineering, Faculty of Engineering, Tohoku-Gakuin University, 1-13-1 Chuo, Tagajo, Miyagi 985-8537 Japan

**Keywords:** Mercury bio-removal, *Yarrowia*, Extracellular polymeric substances, Bio-volatilization, Micro-precipitation, Bioaccumulation

## Abstract

Ecotoxicological implications of mercury (Hg) pollution of hydrosphere require effective Hg-removal strategies as antidote to the environmental problems. Mercury-tolerant yeasts, *Yarrowia* spp. Idd1 and Idd2 strains, were studied for intracellular accumulation and extracellular micro-precipitation of Hg during growth stage of the yeast strains. In a liquid medium containing 870 (±23.6) µg of bioavailable Hg^2+^, 419.0 µg Hg^2+^ (approx.) was taken up by the wet biomasses of the yeast strains after 48 h post-inoculation. Large portion of the adsorbed Hg was found in cell wall (approx. 49–83 %) and spheroplast (approx. 62–89 %). Negligible quantities of Hg were present in the mitochondria (0.02–0.02 %), and appreciable amount of Hg was observed in nuclei and cell debris (15.2–65.3 %) as evidence of bioaccumulation. Extracellular polymeric substances (EPS) produced by the growing *Yarrowia* cells was a complex of protein, carbohydrates and other substances, immobilizing 43.8 (±0.7)–58.7 (±1.0) % of initial Hg in medium as micro-precipitates, while 10.13 ± 0.4–39.2 ± 4.3 % Hg content was volatilized. Transmission electron microscopy coupled with X-ray energy dispersive spectrophotometry confirmed the cellular removal of Hg and formation of EPS-Hg complex colloids in the surrounding bulk solution as micro-precipitates in form of extracellular Hg-nanoparticles. Hg mass balance in the bio-sequestration experiment revealed excellent Hg removal (>97 %) from the medium (containing ≤16 μg ml^−1^ Hg^2+^) by the yeast strains via bioaccumulation, volatilization and micro-precipitation. The yeast strains are also effectively applicable in biological purification technology for Hg contaminated water because of their high self-aggregation activity and separatability from the aquatic environments.

**Graphical abstract:**
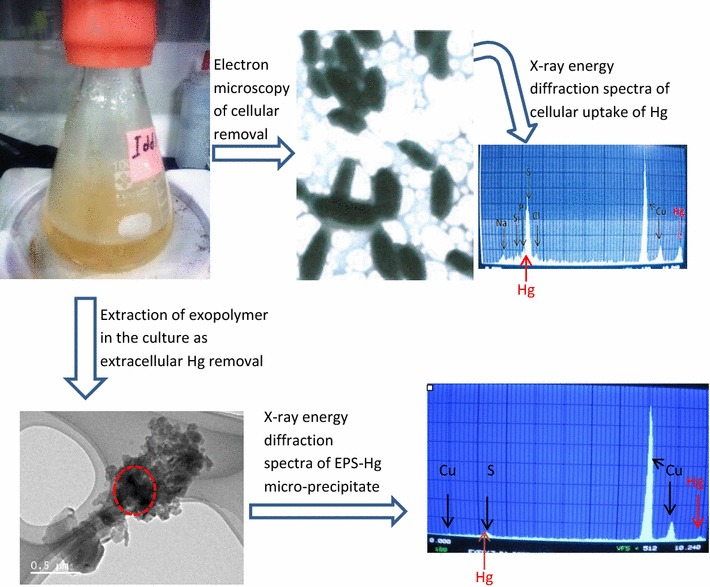
*Yarrowia* species are oligotrophic marine yeasts that exhibited great potentials for mercuric ion remediation technologies, which are classified into four categories based on the process acting on the metal. These include immobilization through biosorption, compartmentation via bioaccumulation, separation from bulk solution via micro-precipitation upon EPS-Hg complex formation, and destruction that is a process to reduce the mercuric ion to metallic mercury.

*Yarrowia* species are oligotrophic marine yeasts that exhibited great potentials for mercuric ion remediation technologies, which are classified into four categories based on the process acting on the metal. These include immobilization through biosorption, compartmentation via bioaccumulation, separation from bulk solution via micro-precipitation upon EPS-Hg complex formation, and destruction that is a process to reduce the mercuric ion to metallic mercury.

## Introduction

Mercury (Hg) pollution has gained worldwide awareness as a major pollutant of the environment. Natural and anthropogenic routes by which this neurotoxic element enters the ecosystems have been widely reported (AMAP/UNEP 2013). High toxicity of Hg is connected to its transport along the trophic levels, transforming to more toxic organic mercury, like the highly neurotoxic methylmercury that causes ‘Minamata disease’ (George 2001; Harada 1972). Antidotes to negative impact of mercury chemical species on public health involve practices that facilitate their removal from contaminated environments.

Mercury (Hg), like other transition metals, is difficult to remove from the environment, because it cannot be chemically or biologically degraded. Remediation of sites polluted with Hg involves processes that target its outright removal, immobilization, or sequestration to innocuous forms. Physiological approaches applied to mitigate Hg pollution in the past were incineration of contaminated samples/materials, chemi-absorption onto chemical compounds, and ion-exchange techniques among others (Hussein et al. 2004). These technologies remain expensive, low specific and environmentally unfriendly. Therefore, biotechnological strategy, whereby microorganisms and their extracellular biopolymers are adopted to removing or immobilizing Hg in polluted system is proposed to be a better remediation option.

Often, abilities of microorganisms to develop survival strategies in polluted systems via phenotypic and genetic modifications are explored in science and technology of decommissioning environmental toxicants. Notable microbial adaptations to toxic metal perturbation that have been variously harnessed in environmental biotechnology of heavy metal mitigation include exclusion of toxic metals via influx and efflux pumping (Nies 2007); partition of toxic metals through adsorption (Oyetibo et al. 2015a), internalization (Strouhal et al. 2003), or sequestration (Huang et al. 1999); and demobilization upon binding with organic ligands like exopolymers secreted by organisms (Guibaud et al. 2009). Microbial techniques currently used in mercury bioremediation include reduction of Hg^2+^ to volatile elemental mercury (Hg^0^) (Huang et al. 1999), and cellular adsorption of mercuric ions using dead and/or live microbial cells (Francois et al. 2012; Oyetibo et al. 2014, 2015a, b). Extracellular polymeric substances (EPS) produced by microbial cells during growth play a crucial role in immobilizing heavy metals (Comte et al. 2008; Francois et al. 2012) and consequently protect cells from deleterious effects of toxic metals.

Applicability of Hg-tolerant yeast strains of the Genus *Yarrowia* for mercury detoxification during growth has been poorly studied, and the role of the exopolymers produced by the yeast strains in Hg immobilization has never been reported based on the literatures available. Previously, two strains of *Yarrowia* spp. (*Idd1* and *Idd2*) that exhibited high tolerance and passive adsorption of mercuric ions (Hg^2+^) were reported (Oyetibo et al. 2015b). It is, therefore, useful to advance exploring the organisms (*Yarrowia* spp. *Idd1* and *Idd2*) for mitigation of mercury pollution in the environment. Hence, this study aimed at investigating extracellular sequestration and immobilization of Hg^2+^, along with cellular accumulation of Hg during the growth of *Yarrowia* strain *Idd1* and strain *Idd2*, for efficient bioremediation of water environments polluted with Hg^2+^.

## Materials and methods

### Culture condition

Stock cells of *Yarrowia* spp. (*Idd1* and *Idd2*), previously isolated and deposited to Japan Collection of Microorganisms under Accession no. JCM 30162 (strain *Idd1*), and JCM 30163 (strain *Idd2*) (stored at −80 °C in 1:1 glycerol:YM broth) (Oyetibo et al. 2015a) were resuscitated by inoculating onto sterile Bacto YM broth (Becton, Dickinson and Co, Sparks, MD, USA). After 72 h incubation (30 °C, 150 rpm), 100 µl culture was spread on dried surface of sterile Bacto YM broth solidified with 1.5 % agar (Wako, Osaka, Japan). The YM broth contained (l^−1^) 5 g, yeast extract; 3 g, malt extract; 5 g, peptone; and 10 g, dextrose. Young and distinct colony (24 h post-inoculation, 30 °C) was inoculated into YM broth (200 ml in 1 l Erlenmeyer flask) and incubated (30 °C; 150 rpm; 48 h).

### Preparation of yeast biomass for mercury bio-removal

Resuscitated yeast strains (approx. 10^6^ cells ml^−1^) were starved overnight in Tris–HCl (0.1 mol l^−1^), and re-suspended in sterile Milli-Q water (previously supplemented with 5.0 µmol l^−1^ HgCl_2_, final concentration) as inocula. Biomass concentrations in cell suspensions were determined as milligram dry weight (mgdw) by drying the aliquot in a pre-weighed aluminium foil container to constant weight at 85 °C overnight.

### Growth dependent cellular removal of mercury

Yeast inoculum (1 ml), as prepared above, was inoculated into 100 ml of YM broth amended with Hg^2+^ (0, 2.0, 4.0, 8.0, 16.0, and 32.0 μg ml^−1^) as HgCl_2_ in 500 ml Erlenmeyer flasks and incubated at 30 °C for 48 h under shaking (150 rpm). Growth of yeast strains was monitored every 6 h as turbidity via optical density at 600 nm (OD_600 nm_) of 5.0 ml sample using a UV-visible spectrophotometer. The blank was uninoculated YM broth supplemented with appropriate Hg concentration. The biomass of each sample was harvested at 10,000×*g* for 10 min, separating the supernatant into a new tube and the pellet was thrice washed with saline solution (0.8 % NaCl), oven-dried at 85 °C overnight and weighed. The culture (before centrifugation), the supernatant and pellets (after centrifugation and washing) were analysed for content of Hg uptake, volatilization (quantitative) and micro-precipitation using a mercury analysis system (as described below). Qualitative Hg(0) volatilization assay was also performed as previously discussed (Oyetibo et al. 2015b) based on the method of Nakamura and Nakahara (1988). Uninoculated YM broth supplemented with appropriate concentration of Hg, and YM without Hg but inoculated with the yeast strains were used as controls.

### Assay for protein

Cells were cultivated in 250 ml YM broth supplemented with 0, 8, and 16 μg ml^−1^ Hg^2+^ as HgCl_2_ (30 °C, 48 h). The cultures were centrifuged (6000×*g*, 30 min, 4 °C), and the supernatants were transferred into new tubes. The pellets were washed thrice with phosphate buffer, suspended in lysis buffer containing 100 mmol l^−1^ NaCl, 50 mmol l^−1^ Tris–HCl (pH 7.5), 1 mmol l^−1^ EDTA, 1 mmol l^−1^ phenylmethylsulfonyl fluoride (PMSF), 0.02 % azide (NaN_3_), 2 % Triton X-100, 10 % glycerol; stirred for 45 min in laden ice slurry, and sonicated (5 × 10 s) in ice bucket. Soluble intracellular protein in supernatant (12,000×*g*, 10 min, 4 °C) was precipitated with (NH_4_)_2_SO_4_ (to yield 80 % saturation), and collected by centrifugation (12,000×*g*, 10 min, 4 °C). Protein pellets were re-suspended gently in 10 ml of buffer containing 20.0 mmol l^−1^ Tris–HCl (pH 7.5), 20 mmol l^−1^ NaCl, 10.0 mmol l^−1^ MgCl_2_; and dialyzed with Biotec symmetric cellulose ester dialysis membrane (8–10 kDa cut off; Spectrum Labs, Rancho Dominguez, CA, USA) at 4 °C against changes of same buffer for 24 h to remove residual (NH_4_)_2_SO_4_. The dialysed suspension was centrifuged (12,000×*g*, 10 min, 4 °C) to pellet insoluble particulate matter, while the supernatant contained protein. The protein was quantified by the method of Bradford (1976), using bovine serum albumin (BSA) (Bio-Rad, Hercules, CA, USA) as standard. Appropriate dilutions of the Coomassie Brilliant Blue G-250 dye (to 1× solution) (Bio-Rad) were prepared according to the manufacturer’s instructions. An 1.0 mg ml^−1^ BSA solution was prepared as standard (approx. absorbance of 0.66 at 280 nm), and a standard curve was created with 5.0 ml of diluted dye added onto 100 µl of BSA concentrations (0.2–0.9 mg ml^−1^) in a dry clean tube in triplicates. To triplicate 100 µl of the sample in tubes, 5.0 ml of diluted dye was added. Absorbance at 595 nm of the vortex mixtures after 5 min, but not later than 1 h, was measured with spectrophotometer and the corresponding value for protein was interpolated from the standard. Reagent without protein was used as blank.

### Isolation of EPS

Methods of Francois et al. (2012) was used in the isolation of EPS from supernatants of 12–48 h (6 h intervals) incubated broth cultures of the yeast strains. The cell pellets collected from 200 ml culture upon centrifugation (6000×*g*, 30 min, 4 °C) were suspended in 10 ml NaCl (20 g l^−1^) solution and centrifuged (6000×*g*, 30 min, 4 °C). The resulting pellets were resuspended in 10 ml NaCl (20 g l^−1^) solution and dialyzed with Biotec symmetric cellulose ester dialysis membrane (8–10 kDa cut off; Spectrum Labs, Rancho Dominguez, CA, USA) against water for 48 h. The dialyzed samples were centrifuged (6000×*g*, 30 min, 4 °C), and the supernatants were freeze-dried and stored at 4 °C. The supernatants of broth cultures of the yeast strains after centrifugation (6000×*g*, 30 min, 4 °C) were pressure-filtered through 0.45 µm pore size Minisart syringe filters (Sartorius Stedim Biotech, Goettingen, Germany). The solutions were kept overnight at 4 °C after treatment with cold 98 % ethanol (ethanol:filtrate, 2:1). The EPS precipitates were recovered by centrifugation (6000×*g*, 30 min, 4 °C). The pellets were resuspended in sterile water, dialyzed against water, lyophilised, and stored at 4 °C.

### Distribution of Hg in cellular components

Cells of 250 ml culture in YM broth supplemented with HgCl_2_ (16.0 μg ml^−1^) were pelleted (3800×*g*, 5 min, 10 °C) yielding about 3 g wet weight of cells. Pellet was washed thrice with sterile potassium phosphate buffer, and resuspended (1 ml g^−1^ of cells) in a solution containing sorbitol (1 mol l^−1^), EDTA (0.1 mol l^−1^, pH 7.5), and lyticase (0.5 mg ml^−1^, 20T, freshly made); and incubated at 37 °C for 60 min. A control was prepared by suspending pellet (1 g) in potassium phosphate buffer (1 ml, pH 7.5). Formation of spheroplast was monitored every 10 min after 30 min post-incubation, by comparing the morphology of cells in the control with those treated with lyticase solution under the microscope (×40 objectives; Zeiss, Overkochen, Germany) at phase contrast mode. Spheroplasts were pelleted (3800×*g*, 5 min, 10 °C) from the cell wall (supernatant), pellets were resuspended (10 ml g^−1^ of cells) in a solution containing Tris–HCl (0.5 mmol l^−1^, pH 7.5), EDTA (1 mmol l^−1^), sorbitol (0.25 mol l^−1^); and incubated (4 °C, 30 min). Nuclei and cell debris were pelleted (2000×*g*, 20 min, 4 °C). The supernatant was transferred to a new tube and centrifuged (15,000×*g*, 20 min, 4 °C) to collect the pellet containing mitochondria. The components comprising cell wall, spheroplast, nuclei and cell debris, and mitochondria were analysed for Hg contents (as explained below) to reflect intracellular distribution of adsorbed Hg. Controls involved cell grown in Hg free medium.

### Analyses of total mercury

The total mercury in EPS-Hg complex (100 µl) was determined directly without any pre-treatment using a fully automated thermal vaporization mercury analysis system, Mercury/MA-3000 (Nippon Instrument Corp., Osaka, Japan). The atomic absorbance of the atomized Hg was measured at a wavelength of 253.7 nm. The instrument was calibrated with standard Hg solution (BDH, Leicestershire, England) at concentrations ranging from 0.1 to 100.0 μg ml^−1^.

### Determination of Hg removal upon self-aggregation activities of *Yarrowia* spp

The need for additional treatment with chemical flocculants to precipitate the yeast cells after uptake of mercuric ions during wastewater management was tested. Common chemical flocculants used in this study include sodium alum, potassium alum, and ammonium alum. Modified method of Nwodo et al. (2014) was used, without addition of 1 % CaCl_2_ and Kaolin suspension. Rather, growth medium supplemented with low and high concentrations of Hg^2+^ (4.0 and 32.0 µg ml^−1^, respectively) were inoculated with the *Yarrowia* spp. strains. After incubation (30 °C, 150 rpm, 96 h), the culture (10 ml) were shared into tubes and each tube was treated with or without 0.5 ml 10 % alum salts solutions. All tubes were vortex-mixed for 30 s and kept still for 5 min. After the aggregation and sedimentation, the upper layer was carefully withdrawn and its OD_600 nm_ was measured and equally analysed for Hg content. A positive control was cultured without Hg amendment, while other controls were alum salts with Hg-amended medium without yeast inoculation, and cultured medium without alum treatment. All the controls were subjected to the same conditions as the main experiments. All experiments were in triplicates and aggregation activity was calculated by using the equation below:

$$\begin{aligned}&{\text{Aggregation}} \;{\text{activity}}\;(supernatant\;Hg\; removal)\; \left( \% \right)\; \\ & \quad \quad \quad \quad = \;\left\{ {\frac{A - B}{A}} \right\}\; \times \;100 \end{aligned}$$

where *A* and *B* are OD_600 nm_ of the positive control and supernatants from samples, respectively.

### TEM and XEDS

Washed cells and EPS suspensions (before and after Hg^2+^ binding) were deposited on copper Formvar-coated electron microscope grids placed on filter paper without staining and allowed to dry. Dried specimens were covered with amorphous carbon film, transmission electron microscope (TEM) micrographs were recorded with a JEM-2000FX II scanning transmission electron microscope (STEM) (JEOL JEM, Tokyo, Japan) equipped with X-ray energy dispersive spectrophotometer (XEDS) operating at an accelerating voltage of 200 kV and a magnification of 29.5 K. The images were acquired with a 2 K (2000 pixel × 2000 pixel) charged-couple device (CCD) camera (Ultrascan 1000; Gatan Inc., Pleasanton, CA, USA). The XEDS detector was used to acquire the X-ray spectra. X-ray mapping was performed using XEDS in conjunction with a STEM module.

### Statistical analyses

All statistical tests including column statistics, row statistics, and non-linear regression (curve fit) of the least squares were performed using the Prism 5 software program (GraphPad Software, San Diego, CA, USA).

## Result

### Growth-time course in relation to Hg bio-volatilization and bioaccumulation

**Fig. 1:**
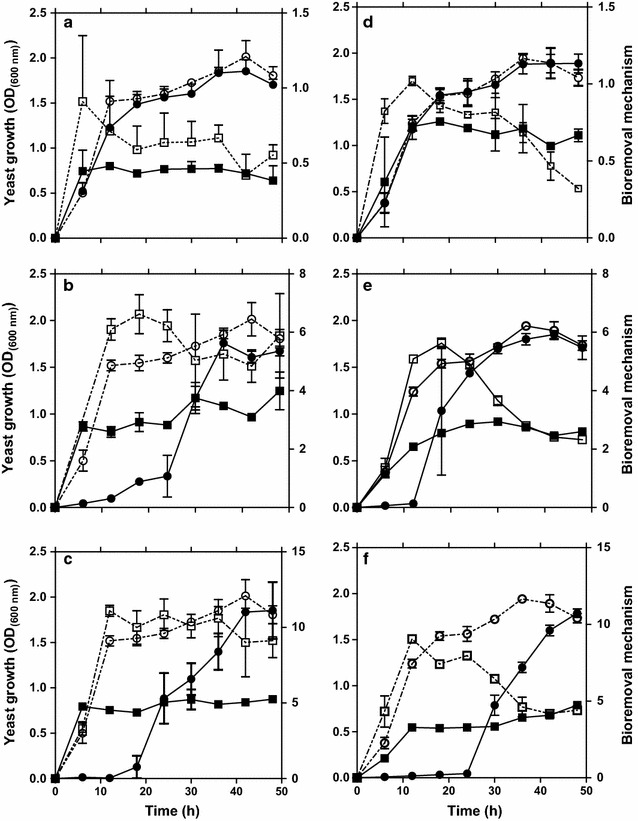
Time-course study of yeast strain *Idd1* (**a**–**c**) and *Idd2* (**d**–**f**) growth in YM broth without HgCl_2_ (*open circle*), and supplemented with HgCl_2_ (*closed circle*), along with Hg sequestration leading to volatilization (*open square*) and cellular accumulation (*closed square*). Growth in medium supplemented with Hg^2+^ concentrations (μg ml^−1^) of 8.0 (**a**, **d**), 16.0 (**b**, **e**), and 32.0 (**c**, **f**) are shown. Temperature: 30 °C, shaking: 150 rpm, and pH: 6.0. *Error bars* represent standard error of mean for triplicate experiments

**Fig. 2:**
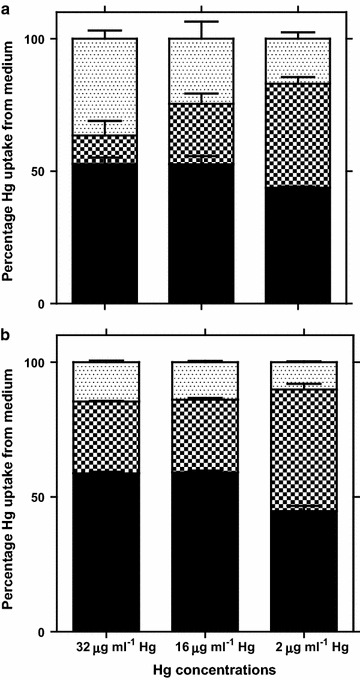
Mass balance of Hg removal by yeast strain *Idd1* (**a**) and *Idd2* (**b**) during growth in medium supplemented with HgCl_2_. Quantity of Hg (expressed as percentages of initial Hg content in medium upon inoculation) remaining in medium as culture supernatant (*shaded bars*), Hg accumulated by biomass (*dark checked bars*), and Hg volatilized (*bright checked bars*). *Error bars* represent standard error of mean for triplicate experiments

The time-course data for Hg^2+^ removal and cellular growth were observed for each strain under ambient pH and temperature conditions prevalent in Lagos lagoon from where the yeast strains were isolated. Figure 1 shows the growth behaviours of *Idd1* and *Idd2* in the media containing different Hg^2+^ concentrations. Growth of *Yarrowia* strains *Idd1* and *Idd2* in medium supplemented with 8.0 mg l^−1^ Hg^2+^ started without a lag phase. The growth pattern was similar to what was observed in a medium that lacked Hg^2+^ amendment (Fig. 1a, d). However, a lag period of ≥12 h preceded exponential growth of *Idd1* and *Idd2* when the growth medium was supplemented with higher Hg concentrations (≥16.0 μg ml^−1^) as shown in Fig. 1b, c, e, and f. There was apparent uptake and volatilization of Hg during lag phase, which did not occur in the control experiment. There were foggy areas on X-ray film (data not included) as confirmation of Hg^2+^ volatilization from Hg^2+^-amended medium after inoculation with *Idd1* and *Idd2*. Hg removal properties of the yeast strains upon Hg depletion in culture due to bioaccumulation and volatilization, in comparison with initial metal concentration in the medium are shown in Fig. 2. Overall, it was observed that 56.2 and 55.3 % of Hg were depleted from medium at low Hg-concentration (2 μg ml^−1^) by *Idd1* (Fig. 2a) and *Idd2* (Fig. 2b), respectively. At higher concentrations (≥16 μg ml^−1^), 47.3 and 41 % of Hg initially present in the culture medium were depleted due to volatilization and bioaccumulation by *Idd1* and *Idd2*, correspondingly.

### Hg micro-precipitation due to EPS complexation with Hg

**Fig. 3:**
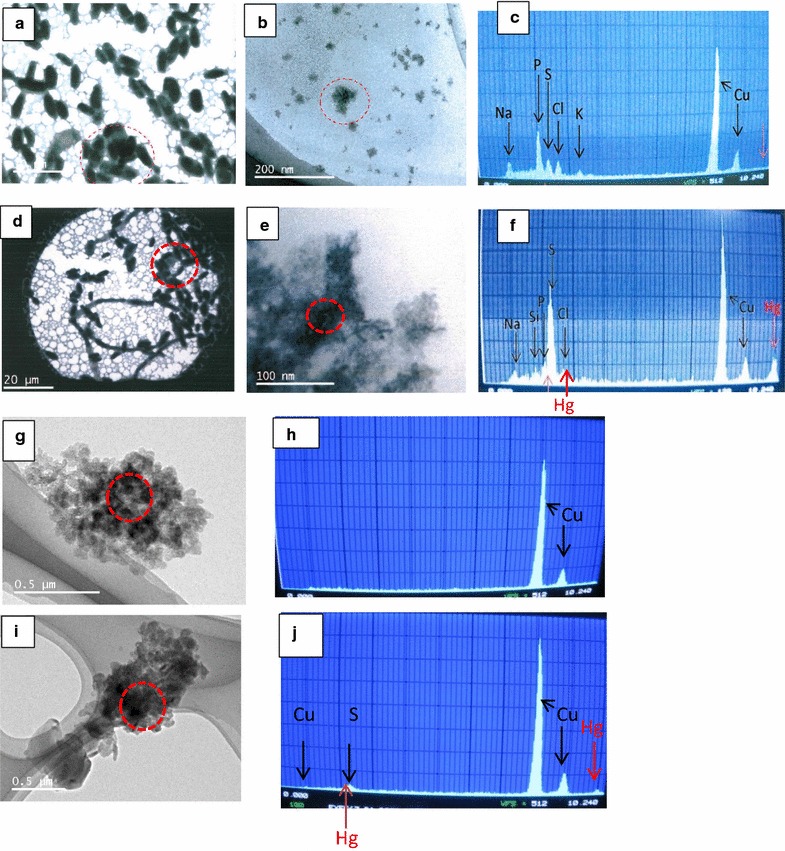
TEM images and XEDS spectra of Hg deposits observed for *Yarrowia* sp *Idd1.* Cells after cultivation in absence of HgCl_2_ (**a**–**c**), cells grown in medium supplemented with HgCl_2_ showing cellular adsorption of Hg (**d**–**f**), extracted EPS of culture in medium without HgCl_2_ (**g**, **h**), and extracted EPS of culture in medium supplemented with HgCl_2_ showing Hg-EPS complex as Hg nanoparticles (**i**, **j**). Areas analysed with XEDS are indicated with dashed oval shapes for cells cultivated without HgCl_2_ (**a**, **b**), cells with HgCl_2_ (**d**, **e**), EPS of culture without HgCl_2_ (**g**), and EPS of culture with HgCl_2_ (**i**) along with their corresponding XEDS spectra obtained for cells without HgCl_2_ (**c**), cells with HgCl_2_ (**f**), EPS from culture without HgCl_2_ (**h**), and EPS from culture with HgCl_2_ (**j**) are shown

Production of EPS was observed in the yeast strains when cultivated in medium supplemented with Hg, and without Hg. Strain *Idd2* produced more EPS than *Idd1*, while more EPS were recovered from the supernatant than from the cell pellets (Table 1). The EPS in both the pellets and supernatants of the culture of medium amended with HgCl_2_ appeared greyish, whilst, the EPS isolated from the culture that lack HgCl_2_ was whitish. Quantification of Hg bound to EPS as evidence of Hg-EPS incorporation and protein contents in EPS were shown in Table 1. More quantities of Hg formed complex with the soluble EPS in the supernatant than cell-bound EPS in relation to amount of EPS produced. Moreover, there was no significant difference between Hg chelated by extracellular EPS from the two yeast strains, unlike the cell bound EPS where *Idd1* relatively bound more Hg than *Idd2*, particularly, at higher Hg concentration (16 μg ml^−1^). The protein content of the EPS increased with increase in Hg(II) concentration in the medium, despite the apparent decrease in the mass of EPS produced in culture with Hg, in relation to culture without Hg exposure. Figure 3 shows the TEM micrographs and X-ray spectra of XEDS examinations of cellular uptake of Hg^2+^, and EPS colloids that formed complex with Hg^2+^, appearing as Hg-nanoparticles upon XEDS analysis.

**Table 1:** Masses, protein quantities, and Hg complexation (precipitation) of EPS extracted from the supernatants and pellets of yeast strains cultivated in media supplemented with or without HgCl_2_

Strain	Hg conc in medium (μg ml^−1^)	Cell bound EPS			Extracellular EPS			
		Mass^a^ (mg dw)	Hg chelated onto EPS (mg gdw^−1^)	Protein in EPS (mg gdw^−1^)	Mass^a^ (mgdw)	Hg chelated onto EPS (mg gdw^−1^)	Precipitated Hg in supernatant (%)	Protein in EPS (mg gdw^−1^)
Idd1	0	0.6	0.209	0.028	3.0	0.035	NA	0.028
	16.0	0.5	2.45	0.707	2.4	8.50	53.1	0.848
	32.0	0.7	4.50	0.752	2.9	13.1	40.8	1.90
Idd2	0	1.2	0.316	0.028	5.0	0.073	NA	0.028
	16.0	0.9	2.59	0.799	2.3	9.41	58.8	0.740
	32.0	1.1	4.46	0.897	1.4	13.8	48.1	1.39

Values represent mean of triplicate experiments

NA means not applicable because the medium was not supplemented with HgCl_2_, but Hg analysis of the culture revealed Hg presence as explained in “Hg micro-precipitation due to EPS complexation with Hg” section. Growth medium (negative control) without inoculation with yeast strains lacks detectable Hg

^a^Mass of EPS extracted from 20 ml yeast culture (OD_600 nm_ = 1.2, approx.)

### Expression of proteins towards Hg toxicity

Periodic extraction and quantification of protein due to response of the yeast strains to Hg toxicity is shown in Table 2. Quantity of protein expressed by the organisms was relative to incubation period and increased with increasing Hg concentration in the system (Table 2) as interpolated with the standard curve from BSA (data not included). At 12 h post inoculation, approximately 0.2–0.8 mg ml^−1^ protein was expressed in comparison with the control culture that only produced 0.1 mg ml^−1^. It was observed that the yeast strains produced protein in response to Hg toxicity, particularly during lag growth phase in medium supplemented with ≥16 μg ml^−1^ Hg^2+^. Moreover, large quantities of protein were produced when the cells were at exponential growth phase, and when the cultures were exposed to Hg. However, a little quantity of protein was produced by cells that were not exposed to Hg^2+^.

**Table 2:** Protein production in response to Hg in growth medium

Yeast strain	Culture age (h)	Hg concentration in growth medium (μg ml^−1^)	Quantity of protein produced (mg ml^−1^)
Idd1	12	0.0	0.094
		8.0	0.604
		16.0	0.668
	24	0.0	0.594
		8.0	0.882
		16.0	0.832
		32.0	0.681
Idd2	12	0.0	0.089
		8.0	0.572
		16.0	0.626
	24	0.0	0.623
		8.0	0.805
		16.0	0.819

### Distribution of Hg(II) in the cellular components of yeast strains

Isolation and analysis of various components of the yeast cells provided distribution of the metal in the cells. Table 3 shows quantities of Hg found in the cell components after 48 h cultivation in YM broth supplemented with approximately 40 µmol l^−1^ HgCl_2_. Total bioavailable Hg in 100 µl growth medium was 870 (±23.6) µg, of which 419 (±156) and 419 (±50.0) µg Hg apparently bound onto intact cells of *Idd1* and *Idd2,* respectively. After removing the cell wall, spheroplast of *Idd1* retained 62.2 % of the bound Hg, while spheroplast of *Idd2* retained 88.5 %. Appreciable quantities of Hg were obviously detected in the components of spheroplast (consisting the nuclei and cell debris), and in the mitochondria (Table 3).

**Table 3:** Distribution of mercury in cells of mercury-resistant yeast strains actively growing in 100 µl YM broth amended with HgCl_2_

Yeast strains	Total mercury in the medium (µg)	Wet weight of cells (mg)	Total mercury bound by cells (µg)	Total mercury bound by cell wall (µg)	Total mercury present in spheroplast (µg)	Total mercury present in nuclei and cell debris (µg)	Total mercury present in mitochondria (µg)
Idd1	871 (23.6)	65.0 (0.5)	419 (156)	203 (9.6)	260 (8.3)	273 (4.5)	10.9 (0)
Idd2	871 (23.6)	67.0 (0.2)	419 (50.0)	347 (38.1)	371 (110.5)	189 (1.3)	9.10 (0.09)

Values in parenthesis are standard error of mean (n = 3)

### Mercury removal upon bio-aggregation

**Fig. 4:**
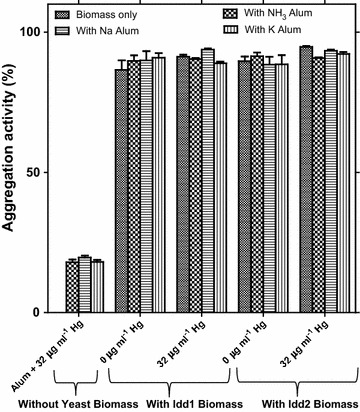
Bio-aggregation activities of *Yarrowia* spp through production of biopolymers alone (self-aggregation) and in combination with chemical flocculants (alum salts) in a medium containing low and high mercury concentrations

The two strains *Yarrowia* spp. investigated in the present study were observed capable of self-aggregating 7 days after inoculation as shown in Fig. 4. The cells were vividly clumped together forming flocs of biomass showing ≥95 % sedimentation without centrifugation. The case in the set-up chemical flocculants vortexed with Hg-amended medium without *Yarrowia* inoculation recorded no sedimentation. Mercury analyses of the supernatants revealed more than 77 % Hg removal efficiency upon self-aggregation activities of the yeasts without any significant difference when chemical flocculants were added to the culture (as shown in Table 4). Interestingly, >94 % Hg initially present in the growth medium supplemented with 32 μg ml^−1^ Hg was observed to be removed after *Idd2* self-aggregation, which was higher than when the chemical flocculants were added (Fig. 4).

**Table 4:** Supernatant Hg removal after aggregation and sedimentation of Hg amended culture medium with/without *Yarrowia* spp. cultivation

Aggregation condition	Supernatant Hg removal (%)
Without yeast biomass (negative control)	
Medium containing 32 µg Hg ml^−1^ with NH_3_ alum salt	17.9 (1.0)
Medium containing 32 µg Hg ml^−1^ with Na alum salt	19.6 (0.7)
Medium containing 32 µg Hg ml^−1^ with K alum salt	18.1 (0.7)
With Idd1 biomass	
Culture biomass (in medium containing 32 µg Hg ml^−1^) without alum salt treatment	91.3 (0.7)
Culture biomass (in medium containing 32 µg Hg ml^−1^) with NH_3_ alum salt	90.4 (0.3)
Culture biomass (in medium containing 32 µg Hg ml^−1^) with Na alum salt	93.8 (0.4)
Culture biomass (in medium containing 32 µg Hg ml^−1^) with K alum salt	88.9 (0.6)
With Idd2 biomass	
Culture biomass (in medium containing 32 µg Hg ml^−1^) without alum salt treatment	94.8 (0.3)
Culture biomass (in medium containing 32 µg Hg ml^−1^) with NH_3_ alum salt	90.8 (0.3)
Culture biomass (in medium containing 32 µg Hg ml^−1^) with Na alum salt	93.4 (0.4)
Culture biomass (in medium containing 32 µg Hg ml^−1^) with K alum salt	92.2 (0.7)

Values in parenthesis are standard error of mean (n = 3)

## Discussion

Living microbial cells can make use of two processes to interact with metals in nature. These include active metabolism in which metals are accumulated inside the cell; and passive metabolism, where metals adhere to surface biomolecules of the cell. Passive adherence of metals onto cell surface is known to be prevalent at lag and early exponential phases, preceding the active bioaccumulation that mostly occurs at late exponential and stationary phases of growth. This phenomenon was observed in our previous study and reportedly dissimilar among the two strains, where passive adherence to Hg^2+^ was weaker with *Idd1* growing cells than those of *Idd2* (Oyetibo et al. 2015b). During lag phase of growth in medium amended with high Hg concentration, the yeast strains (*Yarrowia* spp. *Idd1* and *Idd2*) were apparently overcoming Hg toxicity presumably via extracellular sequestration. A possible explanation to Hg^2+^ transformation during the lag growth phase is likened to Hg^2+^ reduction resulting to volatilization due to activities of biomolecules in the culture and not the chemical constituents of medium or abiotic factors. The reducing biomolecules must have been produced by the yeast cells while adjusting to Hg(II) toxicity prior to growth (lag phase), and during active growth (exponential phase). Recently, *Yarrowia lypolitica* was reportedly able to efficiently remove metals from metals-rich system due to activities of strong reducing agents (Rao et al. 2013). Microbial volatilization of Hg^2+^ has been found to require sulfhydryl compounds (Nakamura and Nakahara 1988).

At stationary growth phase of the yeast strains, endogenous metabolism of available nutrient continues to meet cell reserves for building blocks and energy. It was observed that specific Hg bioaccumulation vividly began during the exponential growth phase, and increased when cells were in stationary phases (Fig. 1). At low metal concentration (8 µg ml^−1^), *Idd2* apparently accumulated more Hg than *Idd1*, but the two strains exhibited very close competences of intracellular Hg-accumulations at higher Hg concentration (≥16 µg ml^−1^). This may be connected to their close relatedness with slight deviance in their phylogeny as suggested in our earlier report (Oyetibo et al. 2015b), coupled with some variances in their physiologies and metabolisms (not yet published). It is obvious that cellular maintenance of the *Yarrowia* strains during stationary growth phase is characterized with transfer of EPS-bound Hg^2+^ or sequestered Hg^2+^ along with nutrients in/out of cells. More so, the cells adjust the osmolality of cell interior as they accumulate more Hg^2+^ from bulk solution. Hg has been reportedly transported across the vacuolar membrane and thereby localised in the vacuoles of *Saccharomyces cerevisiae* (Diffels et al. 2006). While intracellular uptake of Hg^2+^ was going on, it could be inferred that there was dilution of the toxic medium, where mobile Hg^2+^ was incorporated with EPS. This incorporation of Hg^2+^ leads to partitioning of the metals and simultaneous micro-precipitation of mercuric ions. Results of the mass balance during yeast growth in broth supplemented with Hg (Fig. 2), and internalization of Hg apparently indicated that the yeast cells must have performed adsorption and bioaccumulation processes. This involves adherence by surface molecules like proteins, accumulation through helper proteins that cells normally use for the incorporation of essential elements, and/or reduction by enzymatic processes. Cellular density of the yeasts increases as the time passes, providing more available binding sites for Hg^2+^ uptake.

In addition to Hg detoxification strategies (bioaccumulation and volatilization) earlier discussed, another strategy by which the *Yarrowia* strains adopted to demobilize bioavailable Hg in the medium was precipitation. This phenomenon was in terms of EPS-Hg complexation that constitutes an additional Hg removal by the yeast strains to mitigate Hg polluted aquatic system. EPS compounds are matrixes of polysaccharides linked to protein and other biomolecules, which could be ionized. The high Hg content in the greyish cell-bound EPS from HgCl_2_-laden culture corroborates the hypothesis that EPS, as ligand, chelates Hg to form EPS-Hg, whereby Hg is transported into the cellular components through yet to be determined mechanism. Whereas, similar greyish coloured EPS-Hg extracted from culture’s supernatant depicts extracellular precipitation of Hg by the yeasts. In another study, EPS-Hg complexation of the yeast strains has been established to be chemisorption process that is controlled by rate-limiting mechanism (Oyetibo et al., 2016). Greyish colour of the EPS may be due to presence of mercuric compounds, like HgS, emanating from Hg^2+^ sequestration. Precipitations of Hg with sulphide or organosulphur compounds have been identified as tolerance mechanisms in microorganisms (Essa et al. 2005). It is noteworthy that small quantities of Hg were detected in EPS from medium that lacked HgCl_2_ amendment probably due to efflux of intracellular Hg in the inoculum, which was also reflected in the EPS as the cells grew.

Production of EPS is closely linked to binding metal in attempt to alleviate toxic effect of such metal. Due to the presence of charged moieties, EPS ideally serves as natural ligands providing binding sites for other charged particles/molecules including metals (Guibaud et al. 2005). With reference to Fig. 2 and Table 1, it is important that EPS-Hg accounts for bulk of Hg supposedly left in medium as supernatant (*Idd1*: supernatant 53 %, EPS 41 %, unremoved left over 12 %, in medium amended with 32 μg ml^−1^ Hg). It was the same trend in *Idd2*, and more importantly, there were more or less no detectable Hg remaining in the supernatant of culture from medium amended with 16 μg ml^−1^ Hg. This signifies high efficiency of the strains in active detoxification of Hg in polluted aquatic system. Cell-bound EPS have been recognized for their vital role in Hg biosorption (Francois et al. 2012). Nevertheless, EPS act as sink for Hg(II) from point source of pollution, displaying variety of sorption/incorporation sites that influence the transport and fate of Hg in aquatic environment. EPS-Hg complex as soluble precipitants, may get transported to deeper sediments through water column and, therefore, not available to exert toxicity to aquatic lives. Binding and incorporation of metals onto/into EPS have been shown to control the speciation and the distribution of metals in many subsurface and aquatic environments (Lamelas et al. 2006).

Based on TEM-XEDS data of the biomass upon growth in medium supplemented with Hg^2+^, it can be adduced that cellular binding of Hg^2+^ occurs after initial metal incorporation with the cell-bound EPS and neutralization of the chemically active sites. The important deposition of mercury on the EPS as evident in incorporation dynamics and the TEM-XEDS spectra (Fig. 3) suggest that extracellular sequestration and cellular biosorption of Hg by *Yarrowia* spp. would rely on soluble- and bound-EPS, respectively. Increase in the threshold of XEDS spectra for sulphur in relation to Hg incorporation, unlike other elements, indicated that the element must be important during Hg micro-precipitation, suggesting HgS formation via interaction of Hg^2+^ with sulphide (S^2^^−^) moieties of protein molecules of the EPS. Precipitation of mercury with sulphide or organosulphur compounds is a detoxification mechanism that has been described as a mercury tolerance mechanism rather than an effective tolerance process (Glendinning et al. 2005). Similarly, H_2_S produced by sulphate reducing bacteria has been reported as a mercury bioremoval strategy (Essa et al. 2005).

Yeast cells defend themselves against toxic metals by production of specific proteins like metallothioneins, which bind metals in the cytoplasm or transport them into vacuoles. Ordinarily, protein constitutes part of EPS produced by organisms in relation to polysaccharides. Sonication of cells in buffer containing Triton X-100 reduced the potential interference of sugars in the extracts, and consequently optimised the amount of proteins in the extracts. Quantity of protein expressed in response to Hg^2+^ toxicity indicates that certain proteins are involved in response to Hg for intracellular translocation of the metal. Increase in protein expression during fungal response to varying concentrations of heavy metals as tolerance mechanism has been reported (Congeevaram et al. 2007). Strouhal et al. (2003) have also established the involvement of proteins with heavy metal binding, sequestration and bioaccumulation in *Yarrowia* species. The variation in proteome expression patterns among the yeast strains, whereby *Idd1* produced more protein than *Idd2* may probably signify greater functional diversity in *Idd1* (data not shown).

Evidence of bioaccumulation of Hg by *Yarrowia* spp. strains (*Idd1* and *Idd2*) in this study is further strengthened by their distribution of Hg in the cellular components during growth. High level of Hg in the cell wall is probably due to the interaction of the metal with functional groups of the cell wall, particularly the carboxylic groups. The carboxylic groups descended from peptides of the biomolecules, and suggested to be the potential sites for binding Hg(II). A large amount of Hg present in the cell debris confirmed the transport of the metal across the cell wall and membrane into the protoplasm. Distributions of toxic metals within cellular components have been reported to be evidence of metal bioaccumulation (Strouhal et al. 2003).

Aggregates in many biological systems result from microbial and EPS agglutination. EPS are responsible for the cohesive forces that keep components of aggregates together and consequently protect the cells from toxic factors (Miajlovic and Smith 2014). EPS uses bivalent cation, hydrophobic interactions (Yu et al. 2004), van der Waals force and polymer entanglement (Wilen et al. 2003) to link the matrix compounds together. Characteristics of the ability of self-aggregation showed by the yeast strain under Hg^2+^ stress is effective for easy separation of cells and cell-immobilized Hg and then removal of Hg from aquatic solution as evident in Fig. 4.

The Hg bioremoval studies of two mercury-tolerant strains of *Yarrowia* spp. demonstrated extracellular immobilization and sequestration of Hg^2+^ as well as bioaccumulation via distribution of Hg in the cellular components. Soluble extracellular EPS in the medium removed > 43 < 59 % of initial Hg in medium as soluble micro-precipitates, while > 10 < 40 % of Hg content in the medium was volatilized. Overall, the two *Yarrowia* spp. used multi-phenomenal approach to remove more than 97 % of bioavailable Hg^2+^ from the medium (containing ≤16 μg ml^−1^ Hg^2+^) during growth. The yeast strains are also effectively useable in biological purification technology for Hg contaminated water because of their high self-aggregation activity. The two strains of *Yarrowia* are promising biotechnological tools in knowledge-based information required to design a bioreactor for treatment of mercury-laden industrial wastewater and bioremediation of the aquatic environment polluted with mercuric ions.
